# Dynamics of Cellular Responses to Radiation

**DOI:** 10.1371/journal.pcbi.1003513

**Published:** 2014-04-10

**Authors:** Dominik Wodarz, Ron Sorace, Natalia L. Komarova

**Affiliations:** 1 Department of Ecology and Evolutionary Biology, University of California, Irvine, California, United States of America; 2 Department of Mathematics, Rowland Hall, University of California, Irvine, California, United States of America; University of Chicago, United States of America

## Abstract

Understanding the consequences of exposure to low dose ionizing radiation is an important public health concern. While the risk of low dose radiation has been estimated by extrapolation from data at higher doses according to the linear non-threshold model, it has become clear that cellular responses can be very different at low compared to high radiation doses. Important phenomena in this respect include radioadaptive responses as well as low-dose hyper-radiosensitivity (HRS) and increased radioresistance (IRR). With radioadaptive responses, low dose exposure can protect against subsequent challenges, and two mechanisms have been suggested: an intracellular mechanism, inducing cellular changes as a result of the priming radiation, and induction of a protected state by inter-cellular communication. We use mathematical models to examine the effect of these mechanisms on cellular responses to low dose radiation. We find that the intracellular mechanism can account for the occurrence of radioadaptive responses. Interestingly, the same mechanism can also explain the existence of the HRS and IRR phenomena, and successfully describe experimentally observed dose-response relationships for a variety of cell types. This indicates that different, seemingly unrelated, low dose phenomena might be connected and driven by common core processes. With respect to the inter-cellular communication mechanism, we find that it can also account for the occurrence of radioadaptive responses, indicating redundancy in this respect. The model, however, also suggests that the communication mechanism can be vital for the long term survival of cell populations that are continuously exposed to relatively low levels of radiation, which cannot be achieved with the intracellular mechanism in our model. Experimental tests to address our model predictions are proposed.

## Author Summary

The effect of low-dose radiation on cells and tissues is a public health concern, because the human population is exposed to low-dose ionizing radiation coming from a variety of sources, such as cosmic rays, soil radioactivity, environmental contaminations, and various medical procedures. At low doses of radiation, phenomena are observed that do not occur at higher doses, such as radioadaptive responses as well as low-dose hyper-radiosensitivity (HRS) and increased radioresistance (IRR), which are so far not fully understood. Each of these phenomena have been investigated separately, and specific mechanisms have been suggested to explain them. Using mathematical models that are successfully fitted to experimental data under a variety of conditions, we show that a set of basic and documented assumptions about cellular responses to low-dose radiation can explain all three low-dose phenomena, indicating that they are inter-related. According to the model, these phenomena are brought about by the multi-factorial interactions that underlie the population dynamics of the cells involved, and this provides a new framework to understand these responses, and to evaluate the risk to human health posed by exposure to low-dose radiation.

## Introduction

The effect of low-dose radiation on cells and tissues is an important public health topic. The human population is exposed to low-dose ionizing radiation coming from a variety of sources, such as cosmic rays, soil radioactivity, environmental contaminations, and various medical procedures. In order to evaluate health risks posed by low-dose radiation, extrapolations have been made from high-dose data, according to the linear non-threshold model (LNT) [1]. This approach, however, has become controversial because data indicate that cellular responses might differ at low compared to high doses. Examples are phenomena such as radioadaptive responses [2]–[7] as well as low-dose hyper-radiosensitivity (HRS) and increased radioresistance (IRR) [1], [8]–[12], which are so far not fully understood.

Radioadaptive responses are defined as a reduced effect of a radiation challenge following a priming phase of low dose radiation [2]–[7]. They can be observed in the context of several different endpoints, including cell damage, lethality, mutations, and chromosome aberrations. Adaptation is observed in response to both low and high linear energy transfer (LET) radiation, and the exact pattern of adaptation observed is highly variable, depending on factors such as radiation dose, experimental conditions, and the cells/tissues under consideration. The extent to which protection against a radiation challenge is observed can vary significantly. Reduction in the extent of measured endpoints have been found to range between about 5–60% [2]. In addition, the duration of protection varies, and can last a few hours and sometimes longer periods of time after the priming.

The mechanisms underlying radioadaptive responses remain poorly understood [2], [5], [13], [14]. Two basic types of mechanisms can be distinguished [2], [15]. On the one hand, an intracellular response can lead to the adaptation of a cell after it has been exposed to radiation. This involves complex pathways and the temporary induction and suppression of genes. We refer to this state as “memory”, as it is the consequence of a previous radiation hit that leads to protection against future challenges. On the other hand, a cell exposed to radiation can emit signals and induce a state of adaptation in other cells that have not yet been hit by radiation [15]–[17]. Such communication can occur via gap junctions [15], [17]–[20], which is likely to lead to the adaptation of neighboring cells, or through diffusible factors, which can potentially reach cells that are further away from the irradiated cell [15], [17], [21]. Increased levels of reactive oxygen species (ROS) and nitric oxide have been suggested as mediators that contribute to the development of adaptive responses [17], [22]–[24].

This brief summary shows that the development of cellular responses to radiation is the result of complex and dynamical processes that involve interactions of cells within a population. Therefore, mathematical approaches can be useful to complement the large amount of experimental work that is being done and to examine how different mechanisms influence the response of cells to radiation exposure. Here, we construct and analyze mathematical models that examine the effect of the memory and the communication mechanism on cellular responses to radiation. We find that the memory mechanism can not only give rise to the occurrence of radioadaptive responses, but that it can also account for the HRS and IRR phenomena, accurately describing experimental data sets using a variety of cell lines. This suggest that different, seemingly unrelated low dose phenomena might be driven by a common core mechanism. We further find that the communication mechanism can also give rise to the occurrence of radioadaptive responses, indicating redundancy in this context. More importantly, however, the model suggests that the communication mechanism is uniquely required for the long-term survival of functional unaltered cells when cell populations are continuously exposed to low doses of radiation. This has implications for understanding the processes that affect risk in humans that are exposed to certain radiation levels through occupation, such as individuals involved in the aviation industry or in space exploration. Our mathematical results form the foundation for the design of future experimental work that will be needed to address the predictions obtained here.

## Results

### The mathematical models

While our work adds to previous studies that examined mathematical models of radiation responses, e.g. [25]–[35], the models and questions analyzed here are novel. We construct a minimally parameterized mathematical model which describes the processes that can occur in a cell population upon radiation, and track different cell populations, such as healthy cells, hit cells, permanently altered cells, and protected cells. As mentioned above, when cells acquire a permanent change, this can be reflected in a variety of endpoints such as death, mutations, and other aberrations. In the current context we do not aim to distinguish between them and refer to this cell populations collectively as “altered cells”. We model scenarios that correspond to *in vitro* experiments in order to study the basic dynamics of cellular responses to radiation and to relate the theory to available data, which are mostly performed in an *in vitro* setting. We assume that cells do not divide during the time-frame under consideration, because cells with a low turnover rate, such as neural tissue, are likely to be more vulnerable to cell loss and alterations than high turnover tissues, which can compensate with the generation of new cells. In the absence of cell division, predicted dynamics are not dependent on the fate of altered cells, i.e. whether they are damaged and have a lower fitness compared to healthy cells, or whether they disappear due to death. The model includes the following variables: *x* denotes healthy cells that have not been hit by radiation; *y* denotes cells that have received radiation, are altered, and have initiated repair; *w* denotes cells that are adapted and cannot receive permanent damage from radiation; *z* denotes permanently altered cells. The model is formulated as ordinary differential equations that describe the development of cell populations over time:
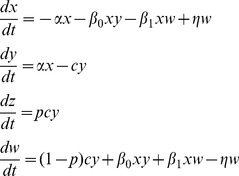
(1)The total number of cells (unaltered+altered) remains constant, since cell division is not assumed to occur. If the result of irradiation (i.e. the permanent alteration) is cell death, then it is more accurate to say that the number of live and dead cells remains constant. This constant population size is given by the initial number of cells in this model. Healthy cells receive a radiation hit with a rate α and attempt to repair the alteration. With a probability *p*, the repair is unsuccessful and the alteration will become permanent. With a probability *(1-p)*, the repair is successful. Upon successful repair, the cell becomes protected from further radiation-induced damage, i.e. it enters the adapted population, *w*. This represents the memory mechanism. With a rate *η*, the adapted cell once again becomes susceptible to radiation. Thus, the average duration of protection is given by 1/*η*. In addition, it is assumed that cell-to-cell communication can lead to adaptation of cells (which represents the communication mechanism). Two modes of communication can occur in the model. A repairing cell, *y*, can induce adaptation in a healthy cell with a rate β*_0_*. Similarly, an adapted cell can induce protection in a healthy cell with a rate β*_1_*. Whether the latter process can be at work is currently not known. If the presence of intracellular factors during repair can allow a cell to bestow protection on a healthy cell, it is possible that these factors remain present in adapted cells, which could theoretically then have the same ability.

We aim to compare the contribution of the two basic pathways by which adaptation can be achieved: (i) The memory mechanism: the intracellular response that allows a repairing cell to “remember” that it was hit and remain protected. (ii) The communication mechanism: the extracellular response whereby cells communicate with each other to induce adaptation. Thus, we construct two models each of which only contains one of the two pathways, and compare their properties.

Keeping with the notation described above, the “memory model” is given as follows:
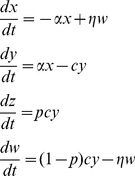
(2)The intracellular response works in the same way as before, i.e. successful repair occurs with a probability *1-p* and leads to protection with the average duration of *1/η*. Communication is assumed to be absent.

The communication model is given by:
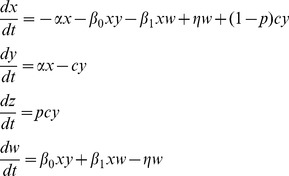
(3)Here, we assume the absence of an intracellular response and no memory. While successful repair happens with a probability 1-p, the cells become fully susceptible again after repair is completed, thus moving to population *x*. Adaptation can only occur through communication, which as before can be mediated by repairing or adapted cells with rates *β_0_* and *β_1_*, respectively.

### Basic dynamics of the memory model

**Figure 1:**
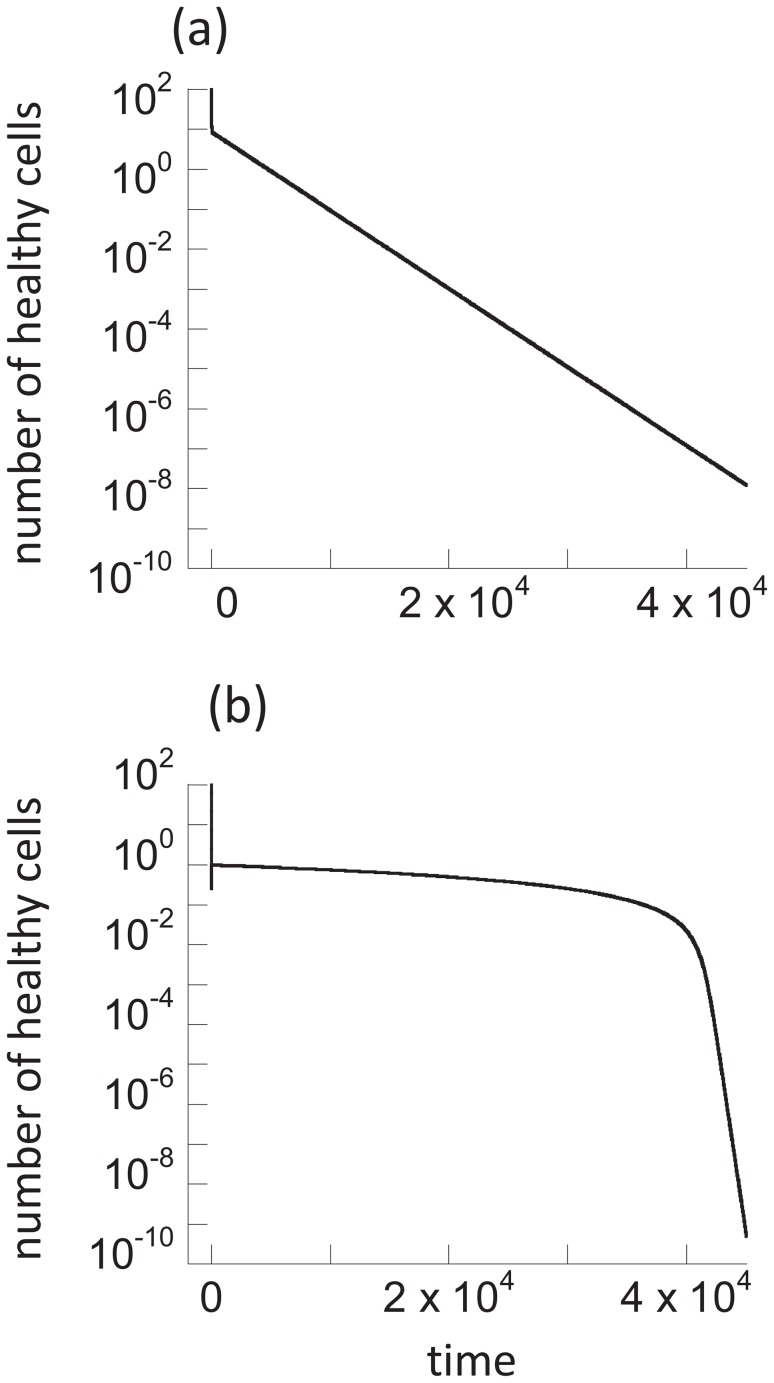
Effect of continuous radiation on the cell population (a) in the memory model (equation 2) and (b) the communication model (equation 3). Note that the long time-spans considered are important to demonstrate the quasi-equilibrium behavior in the communication model, and the absence of this behavior in the memory model. Parameters were chosen as follows (a) *α = 0.1*, *p = 0.05*, *c = 1*, *η = 0.01*, *x_0_ = 100*, *y_0_ = 0*, *z_0_ = 0*, *w_0_ = 0*. (b) *α = 0.1*, *p = 0.05*, *c = 1*, *η = 0.01*, β*_0_ = 10*, β*_1_ = 0*, *x_0_ = 100*, *y_0_ = 0*, *z_0_ = 0*, *w_0_ = 0*.

Here we analyze the properties of the memory model (2). If the population of cells is continuously exposed to radiation in this model, the only long-term outcome is that all cells are permanently altered. That is, an equilibrium is reached where *x = 0*, *y = 0*, *w = 0*, *z = k*. This is not surprising because cells are assumed not to divide and the protection of hit cells is only temporary. Two phases of decline are observed (Figure 1a). A short and relatively fast phase, followed by a slower, long-term phase, brought about by the presence of adapted cells that reduce the effect of radiation on the cell population as a whole. The longer the duration of memory, the slower the decline.

**Figure 2:**
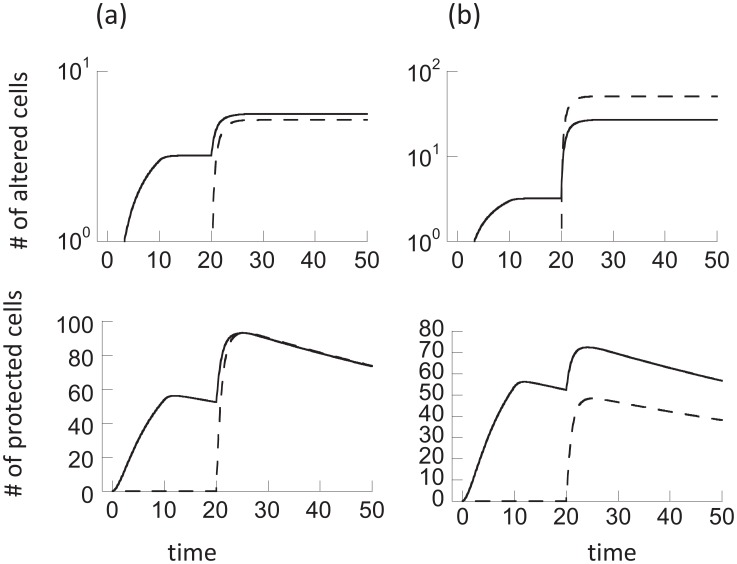
Effect of a relatively large transient radiation phase on the cell population with and without a prior low-dose priming phase in the memory model (equation 2). The solid line is the simulation with low dose priming, and the dashed line is the simulation without low dose priming. Low dose radiation was applied from time unit 0 to 10. Higher dose radiation was applied from time unites 20 to 25. (a) Radiation dose only affects the parameter *α*, i.e. the rate at which cells become hit by radiation. In this case, low dose priming increases the total number of altered cells. Parameters are given by: *α = 0.1* for priming low dose radiation, and *α = 100* for higher dose radiation, *p = 0.05*, *c = 1*, *η = 0.01*, *x_0_ = 100*, *y_0_ = 0*, *z_0_ = 0*, *w_0_ = 0*. (b) Radiation dose affects both parameters *α* and *p*, i.e. it also affects the death rate of hit cells. Now, low dose priming reduces the total number of altered cells, corresponding to a radioadaptive response. Parameters are the same as in (a), with the exception that *p = 0.05* for the low priming dose of radiation and *p = 0.5* for the higher dose.

Next, we examine the effect of a phase of low dose radiation prior to a phase of high dose radiation, the scenario typically discussed in the context of radioadaptive responses. Figure 2 compares the number of altered cells generated by a higher radiation dose following either a phase of lower dose “priming”, or no priming radiation. The impact of the priming radiation depends on what is assumed about the effect of the radiation dose on the model parameters (Figure 2).

Increasing the radiation dose can lead to a higher number of cells that receive genetic damage, which in the model is equivalent to increasing the parameter *α*. If this is the only effect of radiation, a low dose priming radiation cannot improve the outcome (i.e. reduce the total number of altered cells generated). With the priming radiation, the number of altered cells is always higher than in the absence of the priming dose (Figure 2a). The extent of the difference depends on parameters, and the difference can at best be very small such that in practice there is no discernable difference in the number of altered cells generated in the two scenarios.

**Figure 3:**
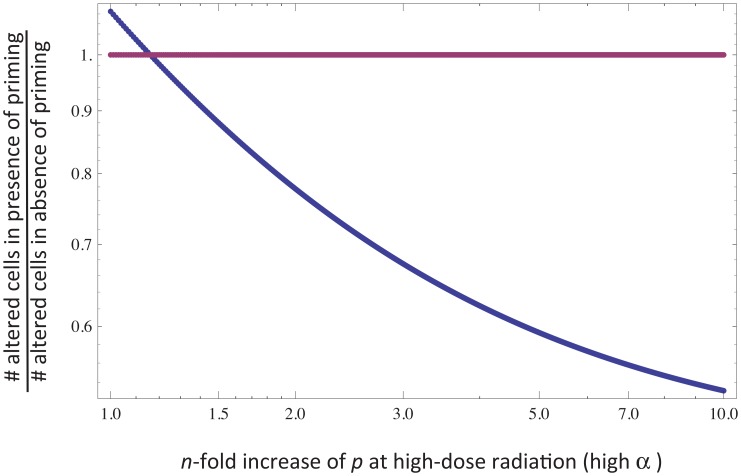
In order for the memory model (2) to reproduce the radioadaptive response, the probability for a cell to become permanently altered by radiation exposure, *p*, must increase sufficiently at the higher radiation dose, *α*. In other words, the radioadaptive response is not observed in the model if the increase in the parameter *p* at the higher value of *α* lies below a threshold. The graph plots the number of permanently altered cells in the presence of priming divided by the number of altered cells generated in the absence of priming, as a function of *n*-fold increase in the value of *p* at high *α*. If the value of *p* for high α is not increased sufficiently, more permanently altered cells are generated in the presence of priming. In contrast, if the value of *p* is increased by a threshold amount at high *α*, then priming lowers the total number of altered cells relative to the scenario where no low-dose priming is given. The horizontal line represents the ratio of one, where priming makes no difference. Base parameters are given as follows: *α = 0.1* for priming low dose radiation, and *α = 100* for higher dose radiation, *c = 1*, *η = 0.01*, *x_0_ = 100*, *y_0_ = 0*, *z_0_ = 0*, *w_0_ = 0*. For low-dose priming, *p = 0.05*. For high dose challenge, the value of *p* is increased *n*-fold, the horizontal axis of the graph.

Now, let us assume that in addition, the radiation dose-rate determines the extent to which each cell on average becomes genetically damaged, and thus the chance of the cell to become permanently altered. That is, increasing the radiation dose also increases the parameter *p*. In this case, low dose priming can lead to a reduction of the total number of altered cells generated (Figure 2b). However, for the radioadaptive response to occur in the model, it is important that the parameter *p* is increased beyond a threshold. If the increase in *p* is less and lies below the threshold, the results are similar to those observed in Figure 2a. This behavior is shown graphically in Figure 3. The more the probability of a hit cell to become permanently altered increases with radiation dose (i.e. the faster the function *p(α)* rises), the stronger the protection that results from the priming dose. A similar, although less pronounced effect is seen if we assume that the duration of protection becomes shorter if the cells are hit with a higher radiation dose. Whether this can occur is unknown, although it could be feasible that a larger extent of genetic damage leads to an impaired protective response by the cells.

These results suggest that a priming dose can only protect against damage by a subsequent, higher dose of radiation if the increase in radiation dose simultaneously affects more than one parameter, such as the fraction of cells that receive a hit and the extent to which each cell becomes damaged. This is probably a realistic scenario, and the memory mechanism can thus likely account for the occurrence of radioadaptive responses.

### Memory model can account for low dose radio-hypersensitivity and increased radioresistance

Studying the survival of cells following a phase of radiation at different doses has been the subject of much investigation. The early notion was that the dependence of cell survival at low doses of radiation could be obtained by extrapolating from the linear-quadratic relationship observed at higher doses. However, when techniques became available to measure cell survival at lower doses of radiation, the situation turned out to be more complex. At very low radiation doses (<0.3 Gy), a heightened sensitivity of cells to radiation has been observed. This is seen as an increased decline slope of cell survival as a function of radiation dose in this low dose range, and is called hyper-radiosensitivity (HRS) [1], [8]–[12], [36], [37]. At slightly higher doses (0.5–1 Gy), a phase of relative radioresistance is observed, characterized by a much reduced decline slope of the survival curve in this dose range. This has been termed increased radioresistance (IRR) [1], [8]–[12], [36], [37]. At higher doses (≫1 Gy), the classical linear-quadratic relationship describes the data on cell survival as a function of radiation dose well [8]. The reasons underlying the occurrence of HRS and IRR are not well understood. Obtaining further insights into these phenomena is important in order to assess the risks of radiation at lower doses. Various molecular processes within cells such as different efficiencies of DNA repair processes at different radiation doses or varying sensitivities of cells to radiation in different stages of the cell cycle have been implicated in explaining HRS and IRR [8]. HRS has been suggested to occur as a result of apoptosis in cells that fail to properly induce repair at low doses of radiation. IRR has been hypothesized to be caused by a change in the G2 checkpoint induction at slightly higher doses of radiation. Mathematically, the complex dependence of cell survival on radiation dose has been well described by Joiner's induced repair model [8], [38], [39], which is a phenomenological extension of the linear quadratic model. While this model provides a good description of the curve, it does not provide an underlying explanation.

Here, we show that the three phases of this relationship (HRS, IRR, and the linear-quadratic dependence) can be explained by the simple memory model (2) described above, without having to evoke any further molecular processes. Our model thus suggests a new hypothesis, which can account not only for the occurrence of radioadaptive responses following low-dose challenges, but also for the occurrence of HRS and IRR. This is explored as follows.

**Figure 4:**
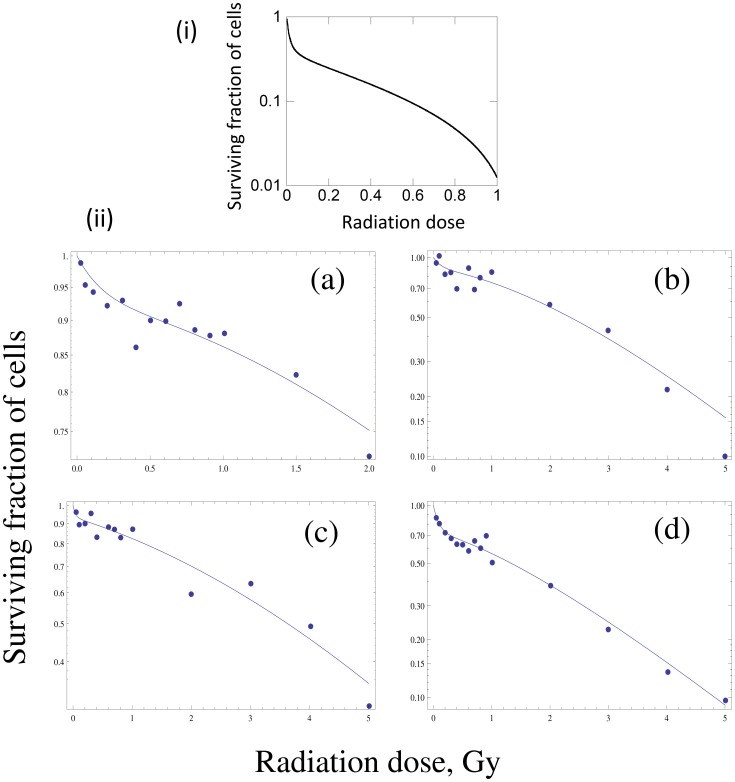
Dose-response curve predicted by the memory model (equation 2). (i) General picture. The fraction of cells surviving after a defined radiation time is plotted against the radiation dose. The model can reproduce experimentally observed patterns, including the phenomena of HRS and IRR at lower doses. Parameters are given by: *p = 0.4+0.55α*, *c = 1*, *η = 0.01*, *x_0_ = 100*, *y_0_ = 0*, *z_0_ = 0*, *w_0_ = 0*. Radiation was applied for a duration of 150 time steps. (ii). Fits of the model to previously published dose-response curves for different cell lines and radiation regimes. The fitting procedures and the estimated parameters are found in the Supporting Text S1. A two-parametric saturating function was used for *p*: 
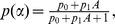
 where 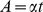. The data were taken from the following sources: (a) p53 mutant T98G cells from reference [41], figure 1; (b) T98G cells from reference [42], figure 1; (c) HGL21 cells from reference [43], figure 1; (d) U138 cells from reference [43], figure 1.

The cumulative radiation dose is proportional to the product *αt*. In model (2) we will first assume that the time-duration is constant, and the dose-rate, *α*, changes. Later we will explore the alternative possibility, where the dose-rate is constant and radiation duration changes. We investigate how the logarithm of the sum of all surviving cells (*ln S = ln (x+y+w)*) after a certain duration of radiation depends on the radiation dose. This can be approximated by the following expression:
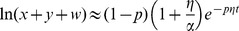
(see Supporting Text S1 for details). The duration of radiation is arbitrary, but the patterns described here do not depend on the specific duration chosen. Figure 4i shows a dose dependency that this model can give rise to. This dependency reproduces the experimentally observed pattern. In plot 4i we assumed that *p* depends linearly on 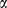. At the lowest radiation doses, we observe the steepest decline of the number of viable cells with increasing radiation doses. This phase corresponds to HRS. This is followed by a much shallower slope of the radiation dose-response curve, as the radiation dose increases, corresponding to IRR. A further increase in the radiation dose leads again to a steeper dependence that is concave down, as seen in the experimental data. Also in accordance with experimental data, the slope of this dependence is not as steep as in the HRS phase.

In order to show that the model not only reproduces the complex dose-response curves on a qualitative level, but that it can describe actual experimental data, we fit the model to specific, experimentally documented dose-response curves, considering different cell types and radiation regimes. To match the experimental conditions, we assumed that the dose-rate was constant, and the radiation duration changed to increase the desired cumulative dose. The model was fit separately to each data set, and parameters were estimated independently. Details of the fitting procedure and parameter estimates can be found in the Supporting Text S1, see also figure caption (Figure 4ii). As shown in Figure 4iia–d, the model fits a variety of data well, demonstrating that it can indeed describe observed biological phenomena. In addition, more data have been successfully fit with the model, which is displayed in the Supporting Text S1.

This shape of the dose-response curve depends on the assumption that the rate at which damaged cells die, *p*, is an increasing function of the radiation dose. The explanation for the shape of this dependence is given as follows. For relatively small radiation doses, not all undamaged cells immediately become hit by radiation. Thus, increasing the radiation dose-rate *α* significantly influences the state of the system as more and more cells become damaged. This determines the slope of the dose-response curve in this parameter regime. For larger values of *α*, all the cells that are in the undamaged cell population, *x*, or re-enter it following repair, become hit by radiation immediately. In this regime, increasing the value of the radiation dose-rate *α* does not change the percentage of fatally hit cells. The only influence of an increased radiation dose occurs through an increased death rate of damaged cells, *p*, since this quantity is assumed to be proportional to *α*. This is what determines the dose-response curve at higher radiation doses.

This theory predicts that the transition from HRS and IRR to the higher dose pattern occurs at a radiation dose when all cells in the population immediately become hit by radiation. In the “low dose regime” the model predicts that not all, but only a fraction of the cells gets instantly hit by radiation, and that this fraction increases with higher radiation doses. In the high dose regime, the model predicts that all cells are hit, and an increase in the radiation dose only influences to what extent each cell is hit. This prediction is supported by previously published experimental data. The number of double strand breaks (DSBs) per cell was measured in MRC-5 cells after 3 minutes of radiation [40]. At low doses, there were less than one DSB per cell, indicating that not all cells received a hit. At higher radiation doses, the number of DSBs per cell rose above one and above 1000 for the highest doses examined. Interestingly, the number of DSBs per cell started rising above one at around 0.05–0.1 Gy. Hence, at this threshold, all cells become immediately hit by radiation and this is predicted to mark the transition from the low-dose behavior to the high-dose behavior. Interestingly, the radiation dose at which typical response curves in mammalian cells transition away from HRS is around this order of magnitude [8], [10] (also seen in Figure 4 and in the Supporting Text S1), although this can vary depending on the cell types and radiation regimes. While this certainly does not prove that this mechanism explains the difference between the dose-response curves at low and high doses of radiation, this warrants further investigation.

Note that the presence of the functional dependence, *p(α)*, introduces an additional level of parameterization in the model. Two remarks are in order in this regard. (1) A memory model with a constant *p* cannot account for the HRS and IRR phenomena. The function *p(α)* must be increasing, and two particular, two-parametric examples of such functions are presented in figure 4 (and in figures 1 and 2 of the Supporting Text S1). (2) Not all increasing functions *p(α)* will lead to the right behavior. In the Supporting Text S1, we present computations pertaining to a general functional form *p(α)*, and discuss the consequences of the different assumptions on the sign and magnitude of the second derivative of *p*.

### Parameter estimates resulting from the data fitting

**Figure 5:**
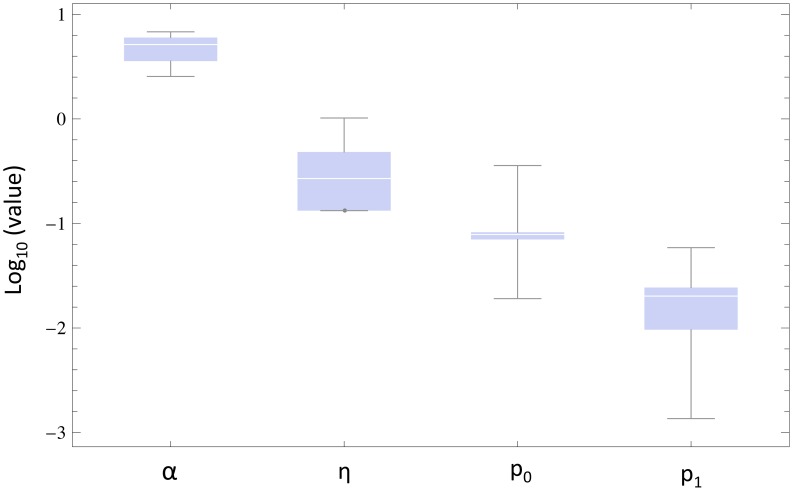
Variability of the parameter estimates across the different experimental setups and cell lines. Parameters 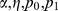 have been estimated by fitting model (2) independently to eight experimental data sets, see Figure 2 of Supporting Text S1 and references therein. Box-and-whiskers diagrams for parameters 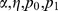 are presented. The dimensionless quantities *p_0_* and *p_1_* parameterize the saturating function 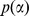, as defined in the caption for figure 4(ii). A box-and-whiskers plot consists of a box that spans the distance between two quantiles surrounding the median, with lines (“whiskers”) that extend to span the full range.

In the previous section, we fit the memory model (2) to a variety of experimental data that documented the radiation dose-response curves in different experimental settings, using a number of different cell lines (Figure 4 and Supporting Text S1). The individual parameter estimates for the different data sets are displayed in the Supporting Text S1, and their variation in the different experiments is discussed as follows. The variability of the parameters from one experiment to the other is shown in Figure 5. This shows that estimates for the parameters *α* and *η* vary relatively little, while estimates for the parameters *p_0_* and *p_1_* vary more extensively. The parameters *α* and *η* determine how many cells in the culture are hit upon radiation, and how long a protected state lasts, respectively. It is interesting that especially the latter parameter remains within relatively narrow bounds across different cell lines and experimental setups. The parameters *p_0_* and *p_1_* determine how a given radiation dose influences the chances for the cell to die (see Figure 5 for further definitions), and the extensive variation in this parameter indicates that this is more cell-type specific.

### Effect of cell-to-cell communication

In this section, the communication model (3) will be studied. The memory mechanism will be ignored in this section in order to investigate which phenomena can be accounted for by the communication mechanism alone. We start again by examining a constant phase of radiation with a specific dose and study the effect on cellular dynamics. As with the memory model, there is only one equilibrium to which the system converges, where all cells are permanently altered, i.e. x^*^ = 0; y^*^ = 0; w^*^ = 0; z^*^ = k. However, some interesting dynamics are observed.

First, assume that communication only occurs between damaged and healthy cells, i.e. β*_1_ = 0*. During radiation, the population of unaltered cells declines towards the equilibrium where all cells are permanently altered, but the pattern of decline can be complex. In some parameter regions, we observe similar decline kinetics as in the memory model, i.e. an initial and relatively short faster phase, followed by a slower, long-term phase due to the presence of protected cells. The rate of decline in this phase is determined by the parameters *p*, *α*, and *η*, in the following way (see Supporting Text S1 for details): (1) if 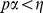, then the decline rate is given by 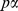, (2) if 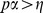, it is given by 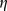. This makes sense: in the former scenario (case (1)), the damage upon irradiation is accumulated slowly (at the rate 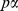), and the transitions between adapted and susceptible states happen relatively fast. Therefore, in the long run, the decline is driven by the direct effect of irradiation. In the latter case (case (2)), the rate-limiting step is to regain susceptibility (at rate 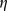), and this is what defines the rate of decline in the long-term phase.

There are, however, two parameter regions, where not two but three phases of decline are observed. These parameter regions are characterized by 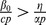
in case (1) above, and by 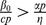 in case (2). In both cases, the inequality requires that the communication of the adapted state among cells happens in an effective way. If this condition is satisfied, the short initial decline phase is followed by a prolonged phase in which the unaltered populations decline only very slowly (Figure 1b). After a certain period of time, the dynamics transition into the final third phase of decline, which is significantly faster, and again determined by the parameters *p*, *α*, and *η*, in the same way as described before. The intermediate, very slow phase of decline (Figure 1b) has not been observed in the memory model and we refer to it as a “quasi-stationary state”. Although the model is not characterized by an equilibrium at which unaltered cells persist, during the intermediate phase, the unaltered cell populations decline so slowly that it can practically be viewed as a long-lived state. The duration of this state is approximately given by 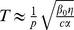 (see Supporting Text S1 for details). These findings are independent of the functional form *p(α)*. Whether a quasi-stationary state is observed for a particular set of parameters will, however, depend on the function *p(α)*, which is discussed in the Supporting Text S1.

The existence of this quasi-stationary state is of biological importance. Although radioadaptive responses are considered mostly in the context of protection against larger doses of radiation following low-dose priming, it can be relevant even in environments that experience continuous exposure to a constant dose of radiation. For relatively long periods of time, the quasi-stationary state allows cell populations without permanent damage to persist at relatively stable levels, while in the absence of a communicable adaptive response, all cells would obtain permanent damage on a short time scale (Figure 1a & b). Similar principles apply if communication only occurs between protected and healthy cells (β*_1_xw*), although it is currently unclear how realistic this assumption is.

**Figure 6:**
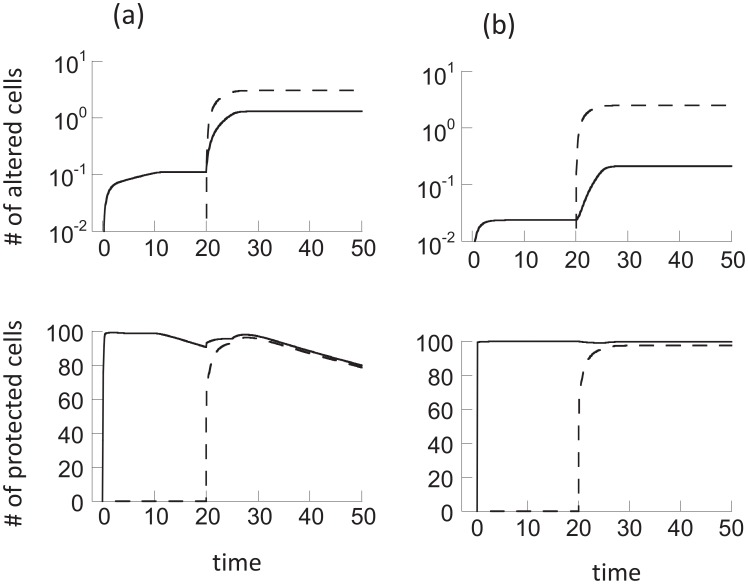
Effect of a relatively large transient radiation phase on the cell population with and without a prior low-dose priming phase in the communication model (equation 3). The solid line is the simulation with low dose priming, and the dashed line is the simulation without low dose priming. Low dose radiation was applied from time unit 0 to 10. Higher dose radiation was applied from time unites 20 to 25. (a) Communication only occurs between hit cells and healthy cells, i.e. β_0_>0, β_1_ = 0. Parameters are given by: *α = 0.1* for priming low dose radiation, and *α = 100* for higher dose radiation, *p = 0.05*, *c = 1*, *η = 0.01*, β*_0_ = 10*, β*_1_ = 0*, *x_0_ = 100*, *y_0_ = 0*, *z_0_ = 0*, *w_0_ = 0*. (b) Communication now also occurs between protected and healthy cells i.e. β_1_>0. Parameters are the same as in (a) except β*_1_ = 1*.

Next, we consider the radioadaptive response in the traditional setting where it is examined how a low dose “priming” radiation phase can protect against a subsequent exposure to high dose radiation. As before, let us first assume that communication only takes place between damaged and healthy cells (β*_1_ = 0*). If the rate of communication is sufficiently fast, we find that low dose radiation can easily confer protection against a subsequent high dose radiation. As shown in Figure 6a, the overall number of permanently altered cells is lower, if high dose radiation is preceded by a low priming dose compared to a scenario where only the high radiation dose is given. The priming radiation triggers the temporary presence of protected cells through communication between hit and healthy cells, and thus the number of cells that can be permanently damaged by the high dose radiation is reduced. In contrast to the memory models, it is sufficient to assume that radiation only affects the number of cells hit, that is, the parameter 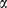. We do not have to assume that as the dose increases, other parameters of the model also change. Therefore, in the communication model, the effect of radioadaptive responses is reproduced under a smaller set of assumptions. The duration for which protection lasts depends on the communication rate and also on the rate at which cells lose their protected status.

Priming can also protect against high dose radiation if communication occurs between already protected and healthy cells (β*_1_>0*). However, there is an important difference: Following the phase of low dose radiation, the population of protected cells is maintained forever and does not decline, as it did in the previous model (Figure 6b). The reason is that the dynamics behave like an infection model: protected cells can “infect” healthy cells with the protected status, and the balance between the generation and loss of protected cells maintains this population at an equilibrium once the priming radiation dose has stopped. Thus, communication between protected and healthy cells can maintain protection for long periods of time without waning (Figure 6b).

In the context of the memory mechanism, we found that the model could account for the HRS and IRR phenomena. In contrast, we found that the communication mechanism cannot explain and describe the HRS and IRR observations in dose-response curves.

### Relative contribution of memory and communication mechanisms

On the one hand, the above analysis has shown that some radiation response phenomena can be explained by only one mechanism, but not the other. Thus, the occurrence of HRS and IRR can arise from the memory mechanism, but not from the communication mechanism. The long-term persistence of unaltered cells in the face of continuous radiation exposure can only be brought about by the communication mechanism, and not by the memory mechanism. In these contexts, the two mechanisms have separate, complementary roles. On the other hand, radioadaptive responses can come about both via communication and memory mechanisms. In this case, the question arises to what extent each mechanism contributes to the occurrence of the radioadaptive response.

This is a difficult question, and not possible to answer with the currently available data. One approach would be to fit the full model (1) to data that document radioadaptive responses. The relative contribution of the two mechanisms can be adjusted in this model by varying the duration of memory protection (large values of *η* lead to short memory and less contribution of this mechanism) and by varying the communication rates β_0_ and β_1_. Alternatively, model (1) could be altered to assume that following a radiation hit, cells fail to induce memory with a probability *p_1_*, and become protected through memory with a probability *1-p_1_*. Varying the parameter *p_1_* would allow us to vary the relative contribution of the memory mechanism, although it is not clear whether this assumption can be justified biologically. However, even if such full models are used to fit data on radioadaptive responses, two important challenges remain: (i) Both mechanisms can independently describe the radioadaptive response. The same time series can be reproduced with different relative contributions of memory and communication by adjusting the remaining parameters of the model. Thus, even if we fit a full model to time series that document radioadaptive responses, this fitting cannot be used to determine their relative contributions. (ii) Data that document the occurrence of radioadative responses do not display the full dynamics of cell populations over time following primary and secondary radiation challenges. They typically display the endpoint following the high-dose challenge, which provides only limited information for model fitting.

In order to determine the relative contributions of memory and communication for the occurrence of radioadaptive responses, new experimental data will need to be generated, and the model will need to be adapted to describe the appropriate experimental conditions. Cellular responses to radiation should be documented under different experimental conditions, and fitting the model to time series of altered and unaltered cells can provide parameter estimates. Cells should be grown in conditions in which they are not in contact with other cells, and in which long-range communication signals are neutralized, thus preventing any communication through gap junctions or emitted signals [15], [17]–[20]. This should be compared to scenarios where cells grow in conditions without contact (no gap junction), but where long range signals for communication are allowed. In a final experimental setup, cells should be allowed to communicate both through gap junctions and through long range signals. To analyze such data, it will also be necessary to study a spatial model of these interactions, in which communication can only occur with nearest neighboring cells, in order to accurately model gap junction communication. Preliminary analysis of such a model suggests that the general conclusions remain robust in the context of the spatial model. That is, there is no significant difference between the effect of gap junction and long-range communication on the general pattern of the dynamics. However, the exact kinetics will differ, which will be important when fitting the model to appropriate experimental data in order to estimate parameters. This is subject to future work, and the current paper prepares the ground for these explorations.

## Discussion

An important message of our analysis is that different, seemingly unrelated phenomena characterizing cellular responses to radiation (low dose hyper-radiosensitivity, increased radioresitance, and radioadaptive responses) might in fact be related and can be explained by the memory mechanism explored in this paper. Another important finding was that our models identified a new mechanism by which cell-to-cell communication can benefit cell populations: During continuous exposure to certain levels of radiation, the communication mechanism gives rise to the existence of a quasi-stationary state, in which the population of functional, unaltered cells only declines with a very slow rate, and practically can remain stable for very long periods of times. Without communication, unaltered cells would decline with a fast rate, which might be detrimental to organisms.

Our analysis was performed under the assumption that cells do not proliferate during the time frame of consideration. This assumption renders cell populations most vulnerable to radiation-induced damage, and this scenario might be particularly important to neural tissue. Organisms are exposed to environmental sources of radiation, and understanding the factors that determine the level of resilience to longer periods of radiation exposure might be especially important for assessing the risk of human occupations that involve increased radiation exposures, such as people working it he aviation industry or involved in space exploration.

Including cell division in this analysis is beyond the scope of the current analysis, but this would generally render the cell populations less vulnerable to radiation. On the one hand, cell proliferation simply opposes the reduction of the cell population. On the other hand, more complex effects could be possible if radiation generates viable but less fit cells, which could be replaced by faster proliferating unaltered cells due to competitive interactions [40]. Such more complicated scenarios can be built on top of the basic interactions described here, which is subject of future work.

## Methods

The cellular dynamics are described by set ordinary differential equations (ODEs), which are constructed in the Results section and subsequently analyzed. Mathematical details that go beyond the scope of the main text are given in the Supporting Text S1. The models are fit to experimental data taken from the literature. Details of the fitting procedures are given in the Supporting Text S1.

## Supporting Information

Text S1 Mathematical details of the models and model fitting procedures discussed in the paper.
(PDF)
