# Supplementary material for: Dynamics of Cellular Responses to Radiation
Source: PLoS Comput Biol. 2014 Apr 10;10(4):e1003513. doi: 10.1371/journal.pcbi.1003513 (PMC3983039; doi:10.1371/journal.pcbi.1003513)
Supplement: Text S1 — Mathematical details of the models and model fitting procedures discussed in the paper. (PDF) [file pcbi.1003513.s001.pdf]

Dynamics of cellular responses to radiation

D. Wodarz, R. Sorace, and N.L. Komarova

Supplementary Information

1 The memory model: the linear-quadratic response and hyper-radiosensitivity

Let us consider the memory model,

$$\dot{x} = -\alpha x + \eta w, \quad (1)$$
$$\dot{y} = \alpha x - cy, \quad (2)$$
$$\dot{w} = (1-p)cy - \eta w, \quad (3)$$

with the initial condition

$$x(0) = 1, \quad y(0) = w(0) = 0.$$

1.1 Analysis

System (1-3) can be written in a matrix form,

$$\dot{\mathbf{x}} = M\mathbf{x},$$

if we set $\mathbf{x} = (x, y, w)^T$ and

$$M = \begin{pmatrix} -\alpha & 0 & \eta \\ \alpha & -c & 0 \\ 0 & (1-p)c & -\eta \end{pmatrix}.$$

Finding the exact solution of this linear problem involves solving cubic equations. Instead, we will use the assumption of the smallness of the parameter η compared to $|c-\alpha|$. The eigenvalues of the matrix M are then given by the following expansion:

$$\lambda_1 = -p\eta + O(\eta^2), \quad (4)$$
$$\lambda_2 = -\alpha - \frac{\eta c(1-p)}{c-\alpha} + O(\eta^2), \quad (5)$$
$$\lambda_3 = -c + \frac{\alpha\eta(1-p)}{c-\alpha} + O(\eta^2). \quad (6)$$

To the first order of η, the corresponding eigenvectors are given by

$$v^{(1)} = \left(\frac{\eta}{\alpha}, \frac{\eta}{c}, 1\right)^T, \tag{7}$$

$$v^{(2)} = \left(1, \frac{\alpha}{c-\alpha} + \frac{\alpha c(1-p)\eta}{(c-\alpha)^3}, -\frac{c(1-p)}{c-\alpha} - \frac{c(1-p)\eta(\alpha^2 - 2\alpha cp + c^2 p)}{\alpha(c-\alpha)^3}\right)^T, \tag{8}$$

$$v^{(3)} = \left(\frac{(1-p)\eta}{c-\alpha}, 1, -(1-p) - \frac{(1-p)\eta(c-\alpha p)}{c(c-\alpha)}\right)^T. \tag{9}$$

The general solution is given by

$$\mathbf{x} = \sum_{j=1}^{3} a_j v^{(j)} e^{\lambda_j t}, \tag{10}$$

and the constants a_1, a_2, and a_3 are found from the initial condition:

$$a_1 = (1-p) - \frac{(c+\alpha)(1-p)(1-2p)\eta}{\alpha c} + O(\eta^2), \tag{11}$$

$$a_2 = 1 - \frac{(c-2\alpha)c(1-p)\eta}{\alpha(c-\alpha)^2} + O(\eta^2), \tag{12}$$

$$a_3 = -\frac{\alpha}{c-\alpha} - \frac{\alpha^2(3c-\alpha)(1-p)\eta}{(c-\alpha)^3 c}. \tag{13}$$

We are interested in the total number of cells,

$$z = x + y + w.$$

Let us explore the dependence of the quantity z on α at a given moment of time.

Let us suppose that $\alpha > \eta$. As $t \gg \max\{1/\alpha, 1/c\}$, we have $\mathbf{x} = a_1 v^{(1)} e^{\lambda_1 t}$, and, neglecting the contribution from y compared to that of x and w, we write

$$z \approx x + w \approx (1-p)\left(1 + \frac{\eta}{\alpha}\right) e^{-p\eta t}. \tag{14}$$

This formula is shown together with the numerical solution of system (13) in figure 1.

Higher dose-rate radiation. Suppose that $\alpha \gg \eta$. Then we have

$$z \approx w \approx (1-p)e^{-p\eta t}.$$

Let us assume that $p(\alpha)$ grows with α. Then the quantity $\ln z$ is a decaying function of α:

$$\frac{d(\ln z)}{d\alpha} = -\frac{(1 + \eta t(1-p))p'}{1-p} < 0.$$

The second derivative is given by

$$\frac{d^2(\ln z)}{d\alpha^2} = -\frac{1}{(1-p)^2}\left((p')^2 - (1-p)(1+\eta t(1-p))p''\right).$$

If p is a linear function of α, or if $p'' < 0$ (which corresponds for example to a saturating function $p(\alpha)$), then $\ln z$ is concave down for all values of α. For p having a quadratic term in its α-dependence, we can have various scenarios, including functions that are concave, convex, and functions with an inflection point.

Intermediate dose-rate radiation. This regime is characterized by $\alpha \sim \eta$, $\alpha > \eta$. Let us assume that $p(\alpha)$ grows with α and consider expression (14). Then the quantity $\ln z$ is a decaying function of α:

$$\frac{d(\ln z)}{d\alpha} = -\frac{(1+\eta t(1-p))p'}{1-p} < 0.$$

The second derivative is given by

$$\begin{aligned}\frac{d^2(\ln z)}{d\alpha^2} &= \frac{1}{\alpha^2(\alpha+\eta)^2(1-p)^2}\left(\eta(2\alpha+\eta)(1-p)^2 - (\alpha(\alpha+\eta)p')^2\right.\\ &- \left.\alpha^2(\alpha+\eta)^2(1-p)(1+\eta t(1-p))p''\right). \qquad (15)\end{aligned}$$

If $p'' \le 0$, this function is concave up as long as

$$\eta(2\alpha+\eta)(1-p)^2 > (\alpha(\alpha+\eta)p')^2,$$

which is satisfied for small values of α, η. A positive second derivative of p can change this result.

Formula (14) breaks down as $\alpha \to 0$. In this regime we need a different approximation, which is described next.

Low dose-rate radiation. If $\alpha < \eta$, we can use an expansion in terms of the small parameter α instead of η. When doing so, we will assume that for small α, we have

$$p(\alpha) \approx p_0 + p_1\alpha.$$

Then the eigenvalues are given by

$$\lambda_1 = -p_0\alpha - \left(\frac{p_0(1+\eta)(1-p_0)}{\eta} + p_1\right)\alpha^2 + O(\alpha^3), \qquad (16)$$

$$\lambda_2 = -\eta - \frac{c\alpha(1-p_0)}{c-\eta} + O(\alpha^2), \qquad (17)$$

$$\lambda_3 = -c + \frac{\eta\alpha(1-p_0)}{c-\eta} + O(\alpha^2). \qquad (18)$$

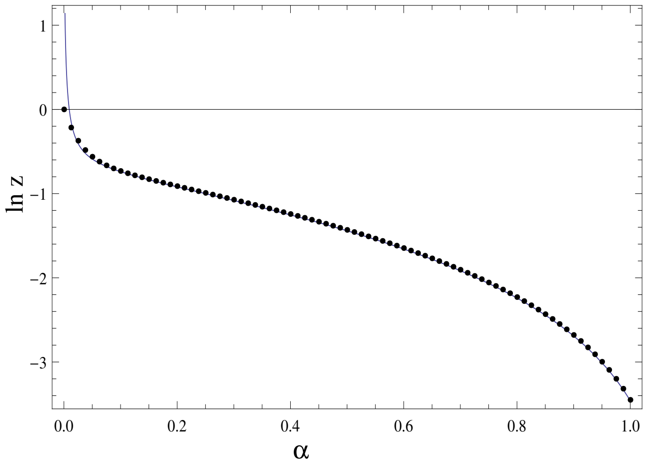

Figure 1: The total number of cells, $\ln z(\alpha)$, has been calculated numerically and plotted as a function of α for $c = 10$, $\eta = 0.01$, $p = 0.4 + 0.55\alpha$, and $t = 50$ (see dotted line). The thin continuous line shows approximation (14).

The eigenvectors are

$$v^{(1)} = \left(1, \frac{1}{c}, \frac{\alpha(1-p_0)}{\eta}\right)^T, \tag{19}$$

$$v^{(2)} = \left(1, \frac{\alpha}{c-\eta}, -1 + \frac{\alpha(cp_0-\eta)}{(c-\eta)\eta}\right)^T, \tag{20}$$

$$v^{(3)} = \left(\frac{\eta(1-p_0)}{c-\eta} + O(\alpha), 1, -\frac{c(1-p_0)}{c-\eta} + O(\alpha)\right)^T. \tag{21}$$

The coefficients in expansion (10) are given by

$$a_1 = 1 - \frac{\alpha(c+\eta)(1-p_0)}{c\eta} + O(\alpha^2), \tag{22}$$

$$a_2 = \frac{c\alpha(1-p_0)}{\eta(c-\eta)} + O(\alpha^2), \tag{23}$$

$$a_3 = -\frac{\alpha}{c} + O(\alpha^2). \tag{24}$$

The solution can be approximated as

$$\mathbf{x} = a_1 v^{(1)} e^{\lambda_1 t}, \tag{25}$$

with a_1, λ_1, and v_1 given by expressions (16, 19, 22). It is possible to show that for this solution, the function $\ln z$ is concave down. Note that this solution becomes valid when solution (14) breaks down. According to formula (14), we have $z > 1$ for $\alpha \to 0$, and solution (25) gives $z \approx 1$ in that regime.

1.2 Intuitive considerations

Let us suppose that $c \gg \eta$. Then we can assume that the variable y is in a quasi-equilibrium, adjusting instantaneously to the level of x:

$$y = \frac{\alpha x}{c}.$$

Using this in equations (1,3), we obtain a system of two equations:

$$\begin{aligned} \dot{x} &= -\alpha x + \eta w, & (26) \\ \dot{w} &= (1-p)\alpha x - \eta w, & (27) \\ x(0) &= 1, & (28) \\ w(0) &= 0. & (29) \end{aligned}$$

This system can be solved easily because it involves a 2×2 matrix. The eigenvalues can be expanded in terms of large α or small η, resulting in the approximate solution (14). Below we outline intuitive considerations which allow us to construct solution (14) for large values of α, without solving system (26-27).

Let us suppose that $\alpha \gg \eta$. At the beginning, the dynamics are dominated by a drop in x and a gain in w:

$$\begin{aligned} \dot{x} &= -\alpha x, & (30) \\ \dot{w} &= (1-p)\alpha x, & (31) \end{aligned}$$

and the solution is $w \approx (1-p)(e^{-\alpha t} - 1)$. This equation states that w starts off at 0 and increases to the level $(1-p)$. The fact that

$$\frac{w}{x} = 1 - p$$

can be seen from the equations, where the term $-\alpha x$ is subtracted from the $\dot{x}$ equation, and only fraction $(1-p)$ of it, $(1-p)\alpha x$, enters the $\dot{w}$ equation.

After this first (fast) phase of decline, slower dynamics follow, where both x and w decay slowly. There, the variable w satisfies the equation

$$\dot{w} = -\eta p w.$$

This can be seen from the quasi-equilibrium solution for x,

$$\begin{aligned} \dot{w} &= \frac{\eta w}{\alpha}, & (32) \\ w(0) &= 1 - p, & (33) \end{aligned}$$

substituted into equation for (27). In other words, the rate of decline for x and w is $p\eta$, and the initial level of w is determined from the previous argument, and equals $1 - p$. Solving system (32-33), we get

$$w = (1-p)e^{-\eta p t}, \quad (34)$$

which explains the behavior for large values of α.

There is a link between the time-scale of the process, t, and the value α. The regime in which the system finds itself depends on the product αt. For relatively small values of α, not all the cells that enter the undamaged compartment (the term ηw in equation (1)) immediately become damaged, and thus increasing α significantly influences the state of the system, as more and more cells become damaged.

For "large" values of α, approximation (34) holds, which corresponds to the system where all the cells that enter the x (undamaged) compartment become damaged. In this regime, increasing the value of α does not change the percentage of damaged cells. The only influence it has is through the value of p, the probability of death.

2 Data fitting

Our model has been fitted to eight data sets from four different papers, [1, 2, 3, 4], where low-dose hyper-radiosensitivity was studied in different cell lines. Because these eight datasets represent eight different cell lines (and not repeats of the same experiment), each dataset was fitted separately, resulting in its own set of best-fitting parameters, see table 1.

In order to reduce the number of fitted parameters, we used system (26-29) as a starting point. Then we also took into account that in typical experiments, the total amount of radiation is varied (rather than intensity). Therefore, we assumed that the probability p is a function of the total radiation amount, $A = \alpha t$. We used the following functional dependency:

$$p = \frac{p_0 + p_1 \alpha t}{p_0 + p_1 \alpha t + 1}.$$

This function changes linearly with the total radiation dose for small doses, and it flattens out as the radiation levels get higher. This function has a meaning of probability, and satisfies $0 \leq p \leq 1$. In order to perform the fitting, we used the software *Mathematica*. Numerical parametric solutions of the ODEs were found and then fitted to the data by using an in-built function, with the following four parameters to fit: η, p_0, p_1, α. Table 1 summarizes the numerical values for the best fitting coefficients. Parameters p_0 and p_1 are unit-less, and the units of both α and η are min^{-1}. We assumed that parameter α, the hit rate of irradiated cells, is proportional to the intensity of radiation, and used the following reported values for the radiation dose rates: 0.51Gy/min for [1], 0.3Gy/min for [2] and [3], and 0.75Gy/min for [4].

Figure 2 presents the results of fitting to eight separate data sets. The fraction of surviving cells was measured as a function of the total radiation dose. The points correspond to the experimental measurements, and the lines to the best fits found with our model. Each plot also contains an inset showing the probability p as a function of the total radiation dose. Out of the eight data sets of figure 2, results for (a), (c), (d), and (e) are presented in the main text.

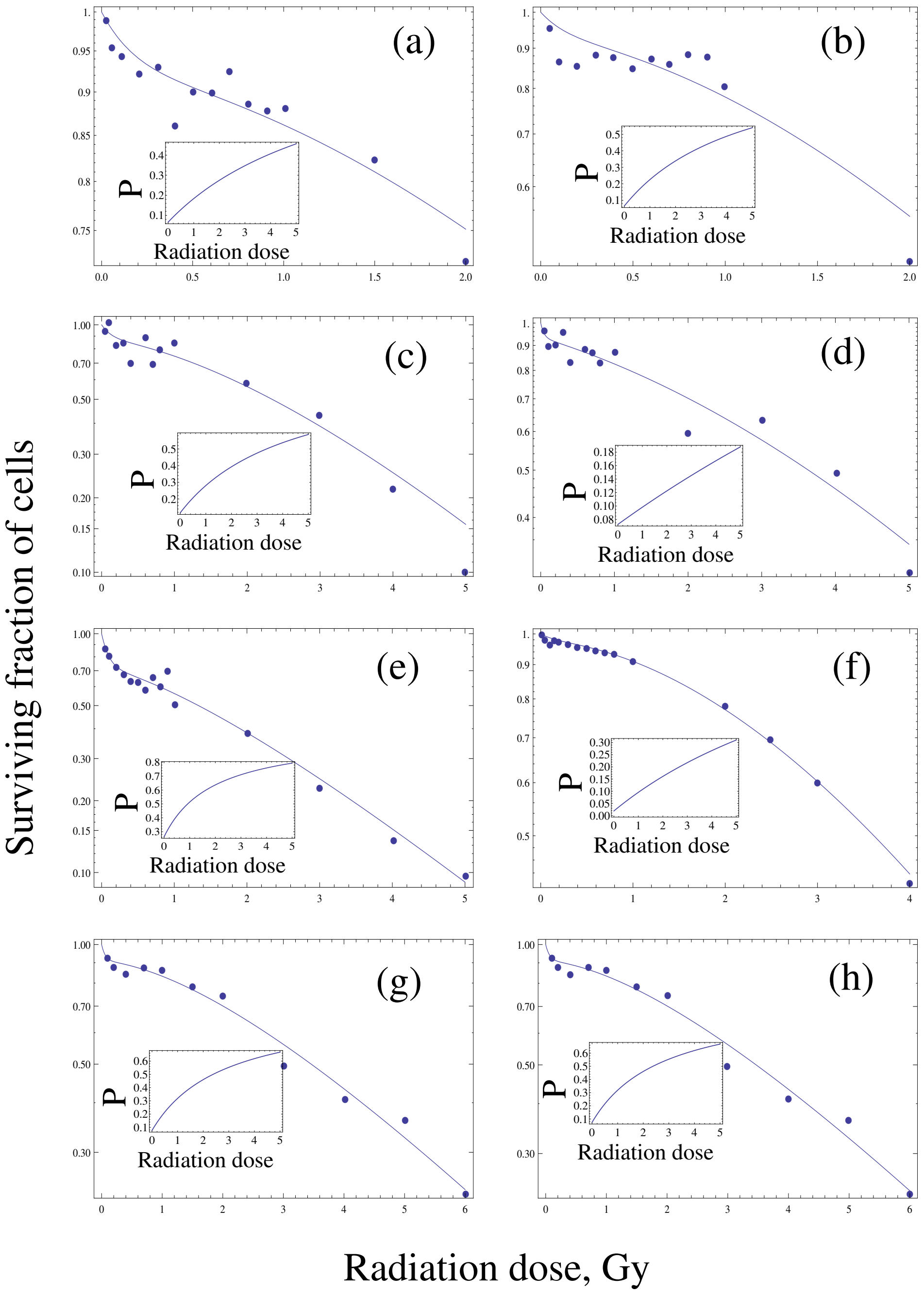

Figure 2: The fitting of the data: (a) and (b) T98G and MR4 cells, both p53 mutant, figure 1 in ref. [4]; (c) T98G cells, figure 1 in ref. [2]; (d) and (e) HGL21 cells and U138 cells, figure 1 in ref. [3]; (f) HT29 cells, figure 4 in ref. [1]; (g) and (h) T98G cells, figures 1 and 2 in ref. [3]. The points represent the measured values, and the lines - the best fit. The insets show the corresponding dependence of the function p on the radiation dose.

Cell type	Reference	α, $\min^{-1}$	η, $\min^{-1}$	p_0	p_1
T98G	[4] fig. 1	5.15	0.48	0.0711	0.0222
MR4	[4] fig. 1	6.81	1.02	0.0703	0.0244
T98G	[2] fig. 1	2.55	0.27	0.133	0.0303
HGL21	[3] fig. 1	6.75	0.47	0.0784	0.00136
U138	[3] fig. 1	3.57	0.20	0.358	0.0587
HT29	[1] fig. 4	4.57	0.76	0.0191	0.00963
T98G	[3] fig. 1	6.00	0.13	0.0825	0.0192
T98G	[3] fig. 2	5.75	0.13	0.0802	0.0202

Table 1: The fitting of the data: (a) and (b) T98G and MR4 cells, both p53 mutant, figure 1 in ref. [4]; (c) T98G cells, figure 1 in ref. [2]; (d) and (e) HGL21 cells and U138 cells, figure 1 in ref. [3]; (f) HT29 cells, figure 4 in ref. [1]; (g) and (h) T98G cells, figures 1 and 2 in ref. [3]. The points represent the measured values, and the lines - the best fit. The insets show the corresponding dependence of the function p on the radiation dose.

3 Communication model: the long-lived survival of cells

3.1 Analysis

Consider the system

$$\dot{x} = -\alpha x + (1-p)cy + \eta w - \beta_0 xy, \tag{35}$$

$$\dot{y} = \alpha x - cy, \tag{36}$$

$$\dot{w} = \beta_0 xy - \eta w. \tag{37}$$

Reductions and rescaling. Let us adopt the quasistationary approximation for the variable y, which requires α and c to be significantly greater than η. We have

$$y = \frac{\alpha x}{c},$$

and the two-equation system is

$$\dot{x} = -p\alpha x + \eta w - \frac{\beta_0 \alpha}{c} x^2, \tag{38}$$

$$\dot{w} = \frac{\beta_0 \alpha}{c} x^2 - \eta w. \tag{39}$$

It is convenient to scale the time-evolution of the system with η, and introduce the following parameter combinations:

$$P = \frac{p\alpha}{\eta}, \tag{40}$$

$$B = \frac{\beta_0 \alpha}{c\eta}. \tag{41}$$

Now the system can be written in a dimensionless form,

$$\begin{aligned} \dot{x} &= -Px + w - Bx^2, && (42)\\ \dot{w} &= Bx^2 - w, && (43)\\ x(0) &= 1, && (44)\\ w(0) &= 0. && (45) \end{aligned}$$

This system goes through several stages of dynamics.

Short time behavior. At the beginning, where w is small, we can ignore the term w in the equation for $\dot{x}$,

$$\dot{x} = -Px - Bx^2, \quad x(0) = 1,$$

and solve the resulting ODE exactly, to give

$$x = \frac{Pe^{-Pt}}{B(1 - e^{-Pt}) + P}. \qquad (46)$$

The initial rate of decline is given by $e^{-(B+P)t}$, which can be obtained by taking the series of this solution in small t. The further dynamics depend on the parameters. There are several cases (denoted by letters **A-E**, which are described below and summarized in figure 3.

Regime A: $P < 1$ and $B \ll P$. In this case, the initial solution (46) can be approximated as $x = e^{-Pt}$, and the corresponding solution for w can be found:

$$w = \frac{B(e^{-t} - e^{-2pt})}{2p - 1}. \qquad (47)$$

It turns out that in this regime, w remains smaller than px, and also $Bx^2 \ll px$. Therefore, the equation for x is simply $\dot{x} = -px$ for the whole duration of time, and solution (47) also holds for all t. The variable x declines at rate P. The long-term decline of w depends on whether P is smaller or larger than $1/2$, and is given by $\min\{1, 2P\}$.

Regime B: $P \ll 1$, $B \gg 1$. In this case, we have a three-phase solution, as described below. Solution (46) holds at the beginning, but as w grows, we achieve a regime where $w \sim Bx^2 \gg px$. In this case, there is an intermediate regime where solution (46) does not hold. Instead, we must look for a slowly-changing solution that satisfies $w \approx Bx^2$. Let us denote $C = w - Bx^2 \ll 1$. In terms of the variables x and C, system (42-43) reads:

$$\begin{aligned} \dot{x} &= -Px + C, && (48)\\ \dot{C} &= -C - 2Bx(-Px + C). && (49) \end{aligned}$$

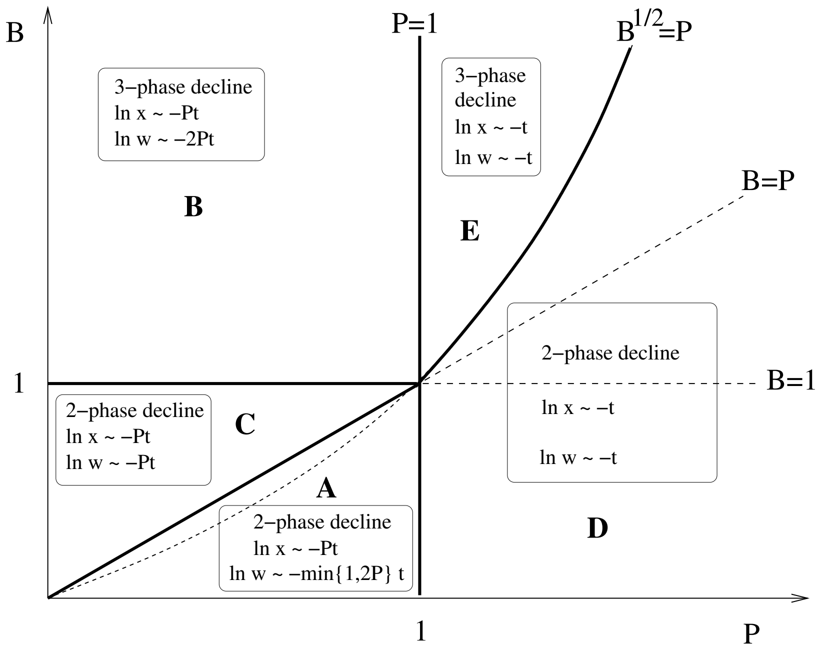

Figure 3: Different behavior of functions x and w. The axes indicate the values of B and P compared to 1 and to each other. Thick solid lines denote region boundaries, such that across these lines the system behavior changes. The regions are marked with letters **A-E**. The text inside each region indicates whether there is a two- or three-phase decline, and also the behavior of the last stage of decay. For example, $\sim -Pt$ means that the corresponding variable decays with rate $p\alpha$; $\sim -t$ means that it decays with rate η; $\sim -\min\{1, 2P\}t$ this means that it decays with rate η if $\eta < 2p\alpha$, and with rate $2p\alpha$ otherwise.

We assume that the variable C changes slowly compared to x and w. Solving the second equation with $\dot{C} = 0$, we obtain

$$C = \frac{2BPx^2}{1 + 2Bx}. \tag{50}$$

An expansion of solution (50) in terms of large B gives

$$C = Px - \frac{P}{2B} + \frac{P}{4B^2 x},$$

and the equation for x becomes

$$\dot{x} = -\frac{P}{2B} + \frac{P}{4B^2 x}.$$

Rewriting this in terms of variables $X = 2Bx$ and $\tau = pt$, we see that

$$\frac{dX}{d\tau} = -1 + \frac{1}{X}, \tag{51}$$

which is parameter-free. The solution of this equation can be found, but we proceed by assuming a short time-scale, that is, $\tau \ll 1$, and by expanding the solution into the Taylor series, $X = A - K\tau$ with $K\tau \ll A$. Using the initial condition $X(0) = 2Bx(0) = 2\sqrt{B}$ (because we assume $x \sim 1/\sqrt{B}$ for this solution), we have

$$-K = -1 + \frac{1}{A}, \tag{52}$$

$$A = 2\sqrt{B}, \tag{53}$$

where the first equation comes from the ODE (51) and the second from the initial condition. Solving this, we obtain

$$x \approx \frac{1}{\sqrt{B}} \left(1 - \frac{2t}{2\sqrt{B}} \right).$$

That is, the scale of slow decay in this regime is given by $p/(2\sqrt{B})$. The approximation found here is valid as long as

$$\frac{Pt}{\sqrt{B}} \ll 1. \tag{54}$$

Once the inequality in condition (54) is reversed, the system enters a long-term regime, where we can assume that

$$w = Bx^2. \tag{55}$$

Substituting this into the equation for $\dot{x}$, we obtain

$$\dot{x} = -Px,$$

which yields $x \propto e^{-Pt}$, and together with equation (55) we obtain $w \propto e^{-2Pt}$.

Regime C: $P \ll 1$, $B \gg P$, and $B \ll 1$. In this case, we have a two-phase decline, as shown below. Just as in the previous case, the system reaches a state where $Bx^2 \sim w \gg Px$. We again transfer to variables x and C and study system (48-49). Expanding expression (50) in terms of small B, we obtain

$$C \approx 2PBx^2,$$

and x satisfies the equation

$$\dot{x} = 2PBx^2 - Px.$$

The general solution of this equation is

$$x = \frac{1}{2B + e^{Pt+\tilde{A}}} \approx ae^{-Pt}$$

(here $\tilde{A}$ and A are constants). We can see that the long-term solution for x decays with a slow rate $P \ll 1$ (and w decays with rate $2p$). In this case, the long-term regime coincides with the intermediate-time regime.

Regime D: $P > 1$ and $\sqrt{B} \ll P$. In this case there are two phases of decline: initially, we have solution (46), which can be approximated as

$$x = e^{-Pt}, \quad w = \frac{B(e^{-t} - e^{-2pt})}{2p - 1} \tag{56}$$

(the latter solution is obtained from substituting the approximation for x into the equation for w and solving exactly). The solution for x given by equation (46) was obtained under the assumption that $w \ll x$. This breaks down because x declines faster than w (condition $P > 1$). Subsequently, a regime is achieved where $Bx^2 \ll Px \sim w$. There are two cases:

- $B \ll P$. In this case we have

  $$w = \frac{Be^{-t}}{2P - 1}, \quad \dot{x} = -Px + w.$$

  Solving the ODE for x, and using the fact that $P \gg 1$, we obtain

  $$x = \frac{Be^{-t}}{2P^2 - 3P + 1}.$$

- $B \gg P$ and $\sqrt{B} \ll P$. In this case, we have

  $$w = e^{-t}, \quad \dot{x} = -Px + w.$$

  Solving the ODE for x, and using the fact that $P \gg 1$, we obtain

  $$x = \frac{Be^{-t}}{P - 1}.$$

In both cases, the solutions for x and w decay with rate 1.

Regime E: $P > 1$ **and** $\sqrt{B} \gg P$**.** This parameter regime is similar to the regime with $P \ll 1$ and $B \gg 1$. Here, we observe three phases. Initially, we have solution (46). Then, we have a regime where $w \approx \beta x^2$ and the approximate rate of decline of x is $P/(2\sqrt{B})$. Finally, both variables decline at rate 1.

3.2 The role of the function $p(\alpha)$

In this section we go back to the original, and not rescaled, values of the system parameters, for an easier biological interpretation.

An important result obtained in the previous subsection is the presence of a long-lived, quasistationary state in the communication model, such that cells can persist for a long time in the presence of a continuous low rate radiation. The existence of such states somewhere in the parameter space is independent of the functional form $p(\alpha)$. Whether or not this effect is observed for a particular set of parameters can be determined by evaluating the inequality

$$\frac{\beta_0}{cp} > \frac{\eta}{\alpha p} \text{ if } p\alpha < \eta, \tag{57}$$

and inequality

$$\frac{\beta_0}{cp} > \frac{\alpha p}{\eta} \text{ if } p\alpha > \eta. \tag{58}$$

hether any of these inequalities hold for a particular value of α will depend on the function $p(\alpha)$, as well as the other parameters.

In figure 4 we present two examples, where all the parameters are fixed, and two different shapes of the function $p(\alpha)$ are studied. Expressions appearing in inequalities (57) and (58) are presented as functions of α. From inequality (57) we obtain $\frac{\eta}{\alpha} > \frac{\beta_0}{c}$, and the function η/α is plotted by a blue dashed line. From inequality (58) we obtain

$$p(\alpha) < \sqrt{\frac{\eta}{\alpha}\frac{\beta_0}{c}},$$

and the left had side of tis inequality is plotted as a black dashed line. Finally, the constant (in α quantity β_0/c is plotted as a horizontal dashed line. These three lines intersect at one point where $\alpha = \frac{\eta c}{\beta_0}$.

Now, for any function $p(\alpha)$ we can check graphically if and when conditions (57-58) hold. Depending on whether $p_1(\alpha)$ is smaller (larger) than the function η/α, we need to check inequality (57) (inequality (58). The first example shown is denoted by $p_1(\alpha)$ and depicts a switch-like function. This function satisfies $p_1(\alpha) < \eta/\alpha$ for α smaller than approximately 0.65 (for the particular parameters chosen in figure 4). Between $\alpha = \frac{\eta c}{\beta_0} = 0.25$ and this value, inequality (57) clearly holds. It also holds for a small interval where $p_1(\alpha) < \eta/\alpha$, until $p_1(\alpha)$ rises above the black dashed line (representing $\sqrt{\frac{\eta}{\alpha}\frac{\beta_0}{c}}$). The set of values of α where the long-term persistence is observed is marked by a thick horizontal line on the α-axis.

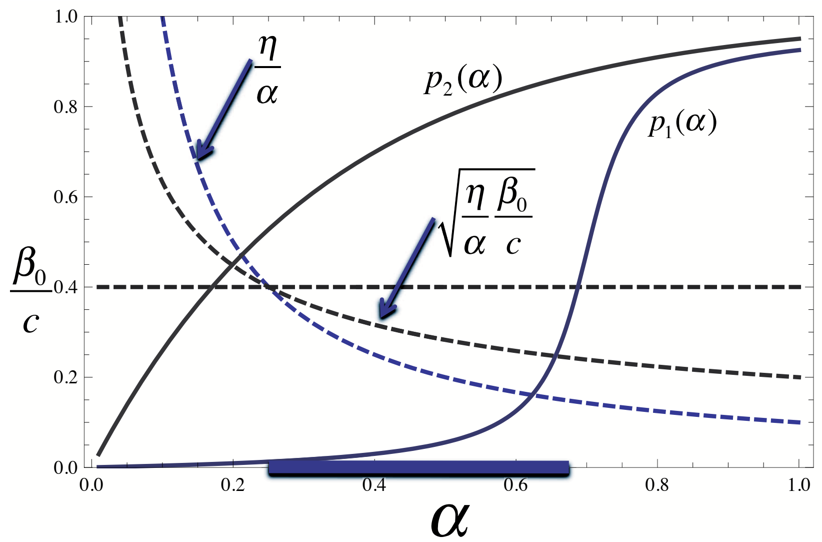

Figure 4: A graphical representation of inequalities (57-58) to show the regions where long-term persistence is observed for the given parameters. Two examples of dependencies of p on α are represented by the two functions $p_1(\alpha)$ and $p_2(\alpha)$. The set of values of α where long-term persistence is observed for function $p_1(\alpha)$ is denoted by a thick line along the α-axis. See text for details. The rest of the parameters are $\beta_0/c = 0.4$, $\eta = 0.1$.

The second example denoted by $p_2(\alpha)$ shows a saturating function of α. For the parameters chosen in figure 4, inequalities (57-58) do not hold for any values of α under the dependency given by function $p_2(\alpha)$. This means that in this case, long-term persistence is not observed.

References

[1] B G Wouters, A M Sy, and L D Skarsgard. Low-dose hypersensitivity and increased radioresistance in a panel of human tumor cell lines with different radiosensitivity. *Radiation research*, 146(4):399–413, 1996.

[2] S Short, C Mayes, M Woodcock, H Johns, and MC Joiner. Low dose hypersensitivity in the t98g human glioblastoma cell line. *International journal of radiation biology*, 75(7):847–855, 1999.

[3] S Short, S A Mitchell, P Boulton, M Woodcock, and MC Joiner. The response of human glioma cell lines to low-dose radiation exposure. *International journal of radiation biology*, 75(11):1341–1348, 1999.

[4] SA Krueger, MC Joiner, M Weinfeld, E Piasentin, and B Marples. Role of apoptosis in low-dose hyper-radiosensitivity. *Radiation research*, 167(3):260–267, 2007.
